# On gene dosage balance in protein complexes: a comment on Semple JI, Vavouri T, Lehner B. A simple principle concerning the robustness of protein complex activity to changes in gene expression.

**DOI:** 10.1186/1752-0509-3-16

**Published:** 2009-01-30

**Authors:** Reiner A Veitia

**Affiliations:** 1 INSERM, U567, Institut Cochin, Paris, France; 2 CNRS, UMR8104, Institut Cochin, Paris, France; 3 Université Paris Descartes, Faculté de Médecine Hôpital Cochin, Paris, France; 4 Université Paris Diderot, Paris, France

## Abstract

A comment on Semple JI, Vavouri T, Lehner B. A simple principle concerning the robustness of protein complex activity to changes in gene expression. BMC Syst Biol. 2008;2:1

## Effects of gene under-expression

I have read with interest the paper by Semple et al. (2008) dealing with the phenotypic effects of protein under- and overexpression as a function of their belonging to protein complexes [1]. Semple et al. 2008 confirmed that, in yeast, genes that inhibit growth when underexpressed often encode subunits of protein complexes. This finding was valid for both core and peripheral subunits. They also reanalyzed overexpression data from an array of yeast strains each one overexpressing a single gene. This array covered 85% of all yeast genes [2]. Semple et al. (2008) find that genes leading to growth defects when over-expressed are not enriched amongst the core or peripheral subunits of protein complexes [1]. Accordingly, they propose a simple principle: "the overall activity of a protein complex is in general robust to an increase, but not to a decrease in the expression of its subunits". The verification of such a simple principle would be more than welcome: at least something simple in biology. With this note, I merely seek to point out places where existing incomplete data leaves untested some hypotheses. Thus, the comment that follows is not a rebuttal of Semple's work.

The first point that attracts the attention in the paper by Semple et al. is the fact that complete gene deletions (when dealing with essential genes or those whose absence leads to a growth defect) are taken as underexpression and not as plain absence of expression. This can be considered as a matter of words but only 'haploinsufficient genes' should qualify as truly underexpressed in their analysis.

In the context of macromolecular complexes an alteration of the stoichiometric balance (i.e. relative amounts) between/among the subunits has been suggested, and often experimentally confirmed, to be harmful and to lead to fitness defects. These alterations result from underexpression (i.e. haploinsufficiency) or overexpression (i.e. in the case of trisomy) of a subunit [3,4]. The proposal of the existence of stoichiometric rules governing the assembly of complexes has been coined as the gene dosage "balance hypothesis" (DBH) [5]. The idea of balance is natural in itself, in the context of complexes, and is also naturally extensible to signaling and transcriptional networks where there are often clearly opposed actors (i.e. a protein kinase "versus" a phosphatase [6]). However, to complicate matters, a complex is most often not isolated but embedded within a cellular (sub)network. Thus, the impact of altering the dosage balance between the subunits can be buffered or, on the contrary, amplified by the relevant network in which the complex in embedded.

Predicting a priori the phenotypic effect of gene underexpression is a difficult task. However, there are cases where strong effects can be expected without taking big risks. For example, if a complex involves identical subunits linked to a common partner (as A in A-B-A) and assembly follows a random pathway (i.e. AB and BA are allowed), a decrease in the concentration of A can lead to a dramatic and non-proportional reduction of trimer yield (<<50% with respect to the wild-type level [3,4]). Even if the complex is embedded in a robust network, such an important decrease of active [A-B-A] might be difficult to buffer. However, halving the amount of subunits present only one time within the complex is expected to lead, in general, to a proportional decrease in the concentration of complex (i.e. a 50% loss). In such cases the effects of underexpression are much less predictable. However, it is clear from the analysis performed by Semple et al. that buffering gene underexpression does not work for a significant proportion of genes encoding subunits of complexes. Unfortunately, whether these subunits tend to be present in multiple copies within the complex or not remains to be explored.

The physicochemical conjecture of the effects of gene underexpression exposed in the context of the trimer A-B-A can be extended to conserved paralogs in the yeast genome (i.e. when their products are functionally interchangeable) [[7] and references therein]. In such instances, the analysis of Semple et al. on essential genes or genes whose deletions induce a growth defect becomes relevant.

## A (statistical) lack of effect of gene over-expression?

The problem experienced by ABA when A is underexpressed can be also figured out as resulting from the relative excess of B. Accordingly, when B is overexpressed in the presence of a normal amount of A there will also be a decrease of ABA (excess of B leads to inactive subcomplexes A-B and B-A). This is also valid for any other complex where B is a bridge between separable subunits or subcomplexes [3,4,8].

In their preliminary analysis (Fig. 1), Semple et al. find that genes leading to fitness defects when overexpressed do not encode subunits of protein complexes more often than expected by random. However, they should recall that the DBH does not predict that overexpression of any type of subunit will lead to a fitness defect. At least as published, the DBH does predict that overexpression of a molecular bridge between two subunits/subcomplexes, in a clearly specified set of conditions, are more likely to decrease the yield of trimer/multimer [3,4,8]. This might also be the case in less straightforward situations such as when there are secondary contacts between the subunits/subcomplexes (such as A-A interactions in ABA, which is now 'triangular' and not linear, see ref. 8). Of course, overexpression of a subunit can be toxic in itself not because the stoichiometry of the complex has been altered but because the monomer contains a domain whose function is autonomous (enzymatic or DNA/RNA binding) and whose excess simply interferes with some metabolic process.

In agreement with the predictions of the DBH, it has been reported that gene pairs encoding interacting subunits tend to have the same number of paralogs (with respect to random expectation), and that genes belonging to huge families rarely encode subunits of complexes [5,9]. These findings can be taken as evidence that overexpression of genes whose products are involved in complexes (and almost certainly in signaling and transcriptional networks) does provoke a disadvantage at an evolutionary scale, whose trace is the selective removal of paralogs of genes involved in complexes or, on the contrary, their co-retention after a whole-genome duplication.

Semple et al. analyze the effects of gene essentiality, haploinsufficiency, overexpression on growth in more details. Specifically, they explore whether genes leading to under- or overexpression phenotypes cluster within complexes. For this, they create bins representing the proportion of subunits in the relevant complexes whose altered expression leads to a phenotype. Concerning their expected distributions, it would be interesting to understand why they are so different according to the types of genes they consider (i.e. essential, haploinsufficient, etc) and why according to the bins under consideration, real protein complexes are overrepresented or underrepresented with respect to random expectation.

From previous discussions it is clear that the types of subunits expected to produce under- and overexpression phenotypes are quite different. Thus, the potential clustering of dosage-sensitive (DS) subunits should depend on the topology (linear, triangular, or other types of spatial arrangements) and the stoichiometry of the complexes analyzed. In short, clustering of DS subunits when underexpressed are more likely to happen in complexes containing several subunits present (in the simplest case) in multiple copies per complex. Clustering of DS subunits when overexpressed is expected for instance in complexes where there are multiple bridges such as in A-B-C-D-E (where B, C and D can be DS). These expected outcomes are just a matter of possibility not of probability (as far as the kinetic and thermodynamic details of the assembly are unknown).

Beyond this theoretical discussion there is a reported clustering of haploinsufficient subunits, which deserves explanation. It is possible that yeast simply needs more than 50% of protein complex to properly work (i.e. low robustness). Thus, halving the amount of any monomer leads to a growth phenotype. Alternatively, and not exclusively, clustering of DS subunits when underexpressed might also be due to a frequent presence of 'repeated' subunits in complexes (i.e. such as A in A-B-A). This may be linked to the fact that the distribution of protein-protein interactions in yeast follows approximately power law [10]. In short, most subunits are poorly connected while only a small number have many partners. This general trend should be valid for protein complexes as well. Thus, most subunits will have, say, 2, 3, 4 protein-protein contacts. Proteins known to establish 2 or 3 contacts can be repeated subunits within the same complex. This is worth being tested by taking into account curated protein-protein interaction data.

Before closing this section it is interesting to explore the limits of the statistical analysis of clustering in the particular case of overexpressed subunits. Let us concentrate on, say, trimers of the type A-B-A or A-B-C. We will assume that, in ALL cases, overexpression of the molecular bridge leads to a fitness defect. This is by far not random and is also predicted by a physicochemical reasoning. Then we assign the values 0 for 'no growth defect' and 1 for 'growth defect' when the subunits are overexpressed. Two thirds of the subunits will be of type 0 (i.e. the 'non-bridge' subunits) and only one third will be 1 (i.e. the bridges). Thus, 100% of our trimers will be in bin 0.33. Indeed, a higher degree of clustering of DS subunits in such trimers is NOT predicted by the DBH. To prepare an expected set, we create random trimers and allow all possible arrangements (000, 010, 100, 111, etc). This random distribution tells that bin 0 contains 29% of random trimers, bin 0.33 contains 44%, 22% will belong to bin 0.66 and the rest to bin 1. What should we conclude from a statistical comparison between the observed and expected distributions? Since 71% of random trimers belong to bin 0.33 or higher do we just 'save' 29% of our trimers (i.e. not expected to occur at random). Given that only bin 0.33 is relevant for our trimers, according to the DBH, do we save 66% of them? Depending on our choice we would conclude that, for trimers, overexpression of a bridge is either a rather minor or a major factor leading to a phenotype when there is overexpression of a subunit.

## An acid test for the DBH

An acid test for the prediction that overexpression can be harmful will be the systematic analysis of the effects of overexpression of, for instance, molecular bridges within the complexes. This criterion is a good startpoint and can/should be relaxed to other subunits that establish links between subcomplexes even if the latter have secondary contacts (see models in ref. 8). This kind of analysis seems to be difficult today because neither the stoichiometry nor the topology are known for a wide variety of complexes. However, this information will be accessible in the future, when crystallographic data for complexes will be available, which will make the aforementioned prediction easily falsifiable. Structural data would only be a first step of the acid test because the simple condition of being a bridge is not really enough. The kinetics of assembly can also play a role (see [6] and especially its supplemental material). For instance, if assembly is ordered/sequential (i.e. first AB, then ABC) overexpression of B has no effect. But even if the kinetics data is not available, the analysis of structural data will drive us closer to the truth. We can make some explorations while waiting. Previous works have shown the existence of high mRNA iso-expression (i.e. similar number of mRNA molecules) for proteins involved in the same complex [5,11,12]. However, there are subunits that are more 'isoexpressed' than others. The concentrations of the former are more likely to be in fine balance. Thus, if one takes mRNA level as a proxy of protein level, one way to go further is to concentrate the analyses on subunits displaying the highest degree of iso-expressivity.

## Some examples of fitness defects linked to gene overexpression

Next I would like to mention several heuristic examples of well-characterized (and not very especially chosen) complexes where overexpression of a subunit is harmful. It should be noted that in several instances the phenotypes are subtler than plain absence of growth.

The yeast genes GPA1, STE4, and STE18 encoding the homologs of the mammalian G-protein α, β, and γ subunits, respectively [13,14] form a linear heterotrimer of the ABC-type (where β is a bridge). This trimer is essential for the transduction of the mating pheromone signal in haploid cells (for the pheromones, the transducer is the dimer βγ itself). As predicted, overexpression of the bridge STE4 alone triggers a response typical of pheromone signal transduction [13]. Of course, co-overexpression of STE4 and STE18 triggers the response as well. These results can be explained by a disruption of the heterotrimer with a concomitant generation of free βγ dimers. Not surprisingly, overexpression of STE18 alone has none of these effects because STE4 is limiting [13]. Since GPA1 is also a promiscuous bridge between the rest of the trimer and membrane receptors, its overexpression leads to diminished signaling due to sequestration of free βγ and probably of the G-coupled receptor that senses the stimulus [15].

The Snf1/AMP-activated protein kinase, involved in stress response such as glucose limitation, provides another example. The Snf1 trimer contains the α catalytic Snf1 subunit, one of three β subunits (Gal83, Sip1 or Sip2) which are bridges and the γ subunit Snf4. Overexpression of the bridge Sip2, decreases the activation of Snf1, not only by disrupting the trimer but also by potentially altering the subcellular localisation of the catalytic Snf1 subunit [16].

We can also explore the complex Sir2/3/4. Sir2 is an NAD-dependent deacetylase involved in chromatin silencing and in telomere position effect in yeast. Silencing is mediated by the Sir heterotrimer, which involves the catalytic subunit Sir2 and the structural proteins Sir3 and Sir4. Recombinant Sir2, Sir3, and Sir4 have been shown to interact with each other directly (with affinities yet to be determined) which leads to a triangular complex [17]. Interestingly, overexpression of Sir4 interferes with silencing and leads to a well-known "anti-SIR" effect [18]. The negative effect of Sir4 on silencing is compensated for by co-overexpression of Sir3 [18]. Upon overexpression of Sir2, Sir3 or Sir4, only increasing Sir3 copy number enhances the telomeric position effect, which suggests that Sir3 is a limiting component of the holo-SIR complex [19]. Overexpression of Sir2 results in a lifespan extension of about 30% (lifespan = the number of cell divisions underwent by the cell before dying) [20]. Thus, the anti-Sir effect of Sir4 overexpression is expected to somehow reduce yeast lifespan (in a way that might escape high-throughput screening?).

Mlc1 is an essential gene that exhibits haploinsufficiency [21]. It interacts with the neck of the essential myosin Myo2 through six IQ motifs of the latter. The complex Myo2/Mlc1 participates in intracellular transport and the structure of the complex Mlc1p-IQ has been solved [22]. Haploinsufficiency of Mlc1 might be explained by the fact that several molecules of Mlc1 are expected to recognize the multiple IQ motifs of Myo2 (which is a dimer itself, [23]). Thus, halving the amount of Mlc1 very likely leads to a dramatic decrease in active Myo2/Mlc complex (<<<50%)! Indeed for multimers such as AnB (where several A monomers interact only with B and n>2), the non-linear effects of either halving A or increasing B sharpen as n increases [8]. Not surprisingly, overexpression of Myo2 is toxic and causes a severe growth defect, which is compensated by overexpressing Mlc1 [21]. The effect of Myo2 overexpression has two alternative and not mutually exclusive explanations. Since each Myo2 molecule plays a role as a bridge by providing 6 IQ sequences for Mlc1, its overexpression will lead to a decreased amount of active complexes through insufficient occupancy of the IQ sites. Moreover, since Myo2 is not the only target of Mlc1 (i.e. Mlc1 also binds to a class II myosin [24]) it may also play a transdominant negative role (i.e. Myo2 titrates Mlc1 molecules required by other myosins) [25].

A transdominant negative effect can also arise when the monomers 'A' can form homo- and heterodimers (i.e. AA and AB) or when they are shared by several partners (i.e. AB, AC, AD, etc). Overexpression of one of the partners will affect the relative concentrations of all dimers. A textbook example is provided by the transcription factors Oaf1 and Pip2. They mediate the induction of genes encoding peroxisomal proteins involved in fatty acid metabolism when oleate is present in the growth medium [26]. Oaf1 may form homodimers that induce the expression of some, but not all oleate-responsive genes, which are fully induced by Oaf1-Pip2 heterodimers [27]. This suggests that Oaf1p alone, most likely in the form of a homodimer, may be recruited to the promoter of a subset of target genes and that the heterodimer may help recognize a larger subset of targets [28]. As expected, overexpression of Oaf1 inhibits fatty acid metabolism by shifting the balance homo-/heterodimer [29].

Interestingly, out of the genes mentioned above, whose overexpression is known to produce a phenotype only STE4/YOR212W appeared in the data used by Semple et al. Just for the sake of the argument, it would be interesting to know how their conclusions would change (or not) if they had access to phenotypic information of the 15% of genes that Sopko et al. did not test.

If protein complexes in yeast cells are pervasively robust to gene overexpression (at least concerning growth and related phenotypes) the challenge will be to move from a 'principle' of robustness to something more mechanistic. On general grounds Semple et al. have proposed some possibilities leading to robustness when a protein subunit is overexpressed. These and other possibilities have also been examined in ref. 30. As a matter of example we can consider how sequential protein binding during complex formation may help avoid dosage effects [4,30]. At least large complexes, such as the ribosome, are assembled in a 'factory line' fashion. A few subunits interact first, some post-translational modifications take place, then more subunits are added to the growing complex, along with more processing, and so on. Accordingly, binding of proteins to the bacterial 16 S rRNA occurs hierarchically, which suggests that early binding proteins organize the binding sites for subsequent proteins. This has been studied in detail for the interactions of proteins S7, S9, S10, and S3, for which binding of one protein strongly enhances recruitment of a subsequent one ([31]and references therein). A similar strategy is expected to be used in the assembly of the eukaryote ribosome. Is this a general rule, which might explain Semple's finding, or a specificity of huge complexes? This remains to be studied.

All in all, Semple et al. did the best they could. Thus, I simply suggest that their results are open to re-interpretation in the future as more experimental data become available.

The mechanisms of phenotypic change in response to increased gene expression

Jennifer I Semple, Tanya Vavouri and Ben Lehner

Whilst it is likely that overexpressing certain specific subunits of certain protein complexes disrupts their assembly and function, global analyses in yeast demonstrate that this cannot be a common explanation for toxicity in response to increased gene expression [32-34]. This does not mean that the mechanism is not important, merely that other, more widespread mechanisms must exist that cause toxicity when genes are overexpressed. It is also clear from the global data in yeast that most subunits of most protein complexes can be overexpressed with no observable phenotypic consequence under laboratory conditions [32-34]. This is still true when considering protein complexes that are performing an essential function in these conditions, and for those where reducing the expression of most subunits is lethal [33]. Where analyzed, overexpression phenotypes are normally different to the loss-of-function phenotypes that result from inhibition of the same genes [34]. Elucidating the molecular mechanisms that cause these gain-of-function phenotypes remains an important challenge for many areas of biology.

## References

[B1] Semple JI, Vavouri T, Lehner B (2008). A simple principle concerning the robustness of protein complex activity to changes in gene expression. BMC Syst Biol.

[B2] Sopko R, Huang D, Preston N, Chua G, Papp B, Kafadar K, Snyder M, Oliver SG, Cyert M, Hughes TR, Boone C, Andrews B (2006). Mapping pathways and phenotypes by systematic gene overexpression. Mol Cell.

[B3] Veitia RA (2002). Exploring the etiology of haploinsufficiency. Bioessays.

[B4] Veitia RA (2003). Nonlinear effects in macromolecular assembly and dosage sensitivity. J Theor Biol.

[B5] Papp B, Pal C, Hurst LD (2003). Dosage sensitivity and the evolution of gene families in yeast. Nature.

[B6] Veitia RA (2005). Gene dosage balance: deletions, duplications and dominance. Trends Genet.

[B7] Saha S, Heber S (2006). In silico prediction of yeast deletion phenotypes. Genet Mol Res.

[B8] Bray D, Lay S (1997). Computer-based analysis of the binding steps in protein complex formation. Proc Natl Acad Sci USA.

[B9] Yang J, Lusk R, Li WH (2003). Organismal complexity, protein complexity, and gene duplicability. Proc Natl Acad Sci USA.

[B10] Hahn MW, Conant GC, Wagner A (2004). Molecular evolution in large genetic networks: does connectivity equal constraint?. J Mol Evol.

[B11] Jansen R, Greenbaum D, Gerstein M (2002). Relating whole-genome expression data with protein-protein interactions. Genome Res.

[B12] Ettwiller L, Veitia RA (2007). Protein coevolution and isoexpression in yeast macromolecular complexes. Comp Funct Genomics.

[B13] Cole GM, Stone DE, Reed SI (1990). Stoichiometry of G protein subunits affects the Saccharomyces cerevisiae mating pheromone signal transduction pathway. Mol Cell Biol.

[B14] Hamm HE (2001). How activated receptors couple to G proteins. Proc Natl Acad Sci USA.

[B15] Dohlman HG, Thorner JW (2001). Regulation of G protein-initiated signal transduction in yeast: paradigms and principles. Ann Rev Biochem.

[B16] Vincent O, Townley R, Kuchin S, Carlson M (2001). Subcellular localization of the Snf1 kinase is regulated by specific beta subunits and a novel glucose signaling mechanism. Genes Dev.

[B17] Moazed D, Kistler A, Axelrod A, Rine J, Johnson AD (1997). Silent information regulator protein complexes in Saccharomyces cerevisiae: a SIR2/SIR4 complex and evidence for a regulatory domain in SIR4 that inhibits its interaction with SIR3. Proc Natl Acad Sci USA.

[B18] Marshall M, Mahoney D, Rose A, Hicks JB, Broach JR (1987). Functional domains of SIR4, a gene required for position effect regulation in Saccharomyces cerevisiae. Mol Cell Biol.

[B19] Renauld H, Aparicio OM, Zierath PD, Billington BL, Chhablani SK, Gottschling DE (1993). Silent domains are assembled continuously from the telomere and are defined by promoter distance and strength, and by SIR3 dosage. Genes Dev.

[B20] Kaeberlein M, McVey M, Guarente L (1999). The SIR2/3/4 complex and SIR2 alone promote longevity in Saccharomyces cerevisiae by two different mechanisms. Genes Dev.

[B21] Stevens RC, Davis TN (1998). Mlc1p is a light chain for the unconventional myosin Myo2p in Saccharomyces cerevisiae. J Cell Biol.

[B22] Terrak M, Wu G, Stafford WF, Lu RC, Dominguez R (2003). Two distinct myosin light chain structures are induced by specific variations within the bound IQ motifs-functional implications. EMBO J.

[B23] Dunn BD, Sakamoto T, Hong MS, Sellers JR, Takizawa PA (2007). Myo4p is a monomeric myosin with motility uniquely adapted to transport mRNA. J Cell Biol.

[B24] Luo J, Vallen EA, Dravis C, Tcheperegine SE, Drees B, Bi E (2004). Identification and functional analysis of the essential and regulatory light chains of the only type II myosin Myo1p in Saccharomyces cerevisiae. J Cell Biol.

[B25] Veitia RA (2007). Exploring the molecular etiology of dominant-negative mutations. Plant Cell.

[B26] Rottensteiner H, Kal AJ, Hamilton B, Ruis H, Tabak HF (1997). A heterodimer of the Zn2Cys6 transcription factors Pip2p and Oaf1p controls induction of genes encoding peroxisomal proteins in Saccharomyces cerevisiae. Eur J Biochem.

[B27] Trzcinska-Danielewicz J, Ishikawa T, Miclalkiewicz A, Fronk J (2008). Yeast transcription factor Oaf1 forms homodimer and induces some oleate-responsive genes in absence of Pip2. Biochem Biophys Res Comm.

[B28] Karpichev IV, Durand-Heredia JM, Luo Y, Small GM (2008). Binding characteristics and regulatory mechanisms of the transcription factors controlling oleate-responsive genes in Saccharomyces cerevisiae. J Biol Chem.

[B29] Smith JJ, Ramsey SA, Marelli M, Marzolf B, Hwang D, Saleem RA, Rachubinski RA, Aitchison JD (2007). Transcriptional responses to fatty acid are coordinated by combinatorial control. Mol Syst Biol.

[B30] Veitia RA, Bottani S, Birchler JA (2008). Cellular reactions to gene dosage imbalance: genomic, transcriptomic and proteomic effects. Trends Genet.

[B31] Stagg SM, Mears JA, Harvey SC (2003). A structural model for the assembly of the 30S subunit of the ribosome. J Mol Biol.

[B32] Gelperin DM, White MA, Wilkinson ML, Kon Y, Kung LA, Wise KJ, Lopez-Hoyo N, Jiang L, Piccirillo S, Yu H (2005). Biochemical and genetic analysis of the yeast proteome with a movable ORF collection. Genes Dev.

[B33] Semple JI, Vavouri T, Lehner B (2008). A simple principle concerning the robustness of protein complex activity to changes in gene expression. BMC Syst Biol.

[B34] Sopko R, Huang D, Preston N, Chua G, Papp B, Kafadar K, Snyder M, Oliver SG, Cyert M, Hughes TR (2006). Mapping pathways and phenotypes by systematic gene overexpression. Mol Cell.

